# Fabrication of Au–Ag Bimetallic Nanoparticles Using Pulsed Laser Ablation for Medical Applications: A Review

**DOI:** 10.3390/nano13222940

**Published:** 2023-11-13

**Authors:** Muidh Alheshibri

**Affiliations:** General Studies Department, Jubail Industrial College, P.O. Box 10099, Jubail Industrial City 31961, Saudi Arabia; heshibri@rcjy.edu.sa or alheshibri.muidh@gmail.com

**Keywords:** gold, silver, bimetallic nanoparticles, pulsed laser ablation

## Abstract

In recent years, the synthesis of Au–Ag bimetallic nanoparticles has garnered immense attention due to their potential applications in diverse fields, particularly in the realm of medicine and healthcare. The development of efficient synthesis methods is crucial in harnessing their unique properties for medical applications. Among the synthesis methods, pulsed laser ablation in a liquid environment has emerged as a robust and versatile method for precisely tailoring the synthesis of bimetallic nanoparticles. This manuscript provides an overview of the fundamentals of the pulsed laser ablation in a liquid method, elucidating the critical factors involved. It comprehensively explores the pivotal factors influencing Au–Ag bimetallic nanoparticle synthesis, delving into the material composition, laser parameters, and environmental conditions. Furthermore, this review highlights the promising strides made in antibacterial, photothermal, and diagnostic applications. Despite the remarkable progress, the manuscript also outlines the existing limitations and challenges in this advanced synthesis technique. By providing a thorough examination of the current state of research, this review aims to pave the way for future innovations in the field, driving the development of novel, safe, and effective medical technologies based on Au–Ag bimetallic nanoparticles.

## 1. Introduction

The applications of metal nanoparticles encompass a wide array of fields, including biomedicine with applications in drug delivery and magnetic resonance imaging contrast agents. They also find utility as catalysts in environmental remediation [1,2] and renewable energy systems [3,4]. In addition, nanoparticles play a pivotal role in photo- and electrocatalytic processes [5,6], such as water splitting [7], and contribute to innovations in solar nanofluids [8], property enhancement, and additive manufacturing for smart materials [9]. Furthermore, metal nanoparticles have been instrumental in the development of food packaging materials with antibacterial properties [10], particularly within the food industry.

Among metal nanoparticles, gold, silver, and their composite materials at the nanoscale have garnered significant interest due to their utility in single electron transistors [11], gas sensors [12], bio detectors [13,14], and catalytic processes [15]. Bimetallic nanostructures, which combine both gold (Au) and silver (Ag), have emerged as superior materials. Their distinctiveness lies in the ability to precisely control their surface plasmon resonance (SPR) due to the collective oscillation of valence electrons within their partially filled electron shells. When subjected to an external field, the valence electrons of AgNPs become excited, resulting in heightened plasmonic properties compared to AuNPs. This phenomenon leads to enhanced catalytic properties, magnetic and optical polarization, electrical conductivity, and antimicrobial characteristics in the bimetallic nanostructures [16].

The synthesis of Au–Ag bimetallic nanoparticles encompasses a wide array of techniques, with two predominant approaches being top-down and bottom-up methods. Top-down approaches such as mechanical milling and chemical etching involve the reduction of bulk materials to nanoscale dimensions [17], often through mechanical, chemical, or physical processes. These methods provide excellent control over particle size and morphology, making them suitable for applications requiring precise engineering. Conversely, bottom-up approach such as sol-gel process involve the assembly of atoms, molecules, or nanoparticles to construct nanomaterials from the ground up providing remarkable control over material composition and atomic arrangement [18], and facilitating the synthesis of complex nanostructures with tailored properties.

The importance of nanomaterials in diverse applications has been constrained by the imperative of producing nanomaterials devoid of contaminants, despite the extensive reports of nanomaterial synthesis over the past century. Among the various synthesis techniques, pulsed laser ablation in liquid (PLAL) has emerged as a powerful tool for generating nanomaterials. Laser ablation involves the irradiation of a target material with a high-energy laser beam, resulting in the generation of vaporized material that subsequently condenses to form nanoparticles. This method offers advantages such as high purity, minimal contamination, and precise control over nanoparticle size and composition, making it a prominent choice in the realm of nanomaterial synthesis.

In this brief perspective article, we provide an overview of how Ag/Au bimetallic nanoparticles are produced using the PLAL method. We commence this review by elucidating the fundamentals of PLAL. We then provide an overview of how laser parameters and the surrounding environment affect the size and structure of the Ag/Au bimetallic nanoparticles that are generated. We also discuss their potential medical applications and address the challenges that must be addressed to enhance their performance.

## 2. The Basics of Pulsed Laser Ablation in a Liquid Environment

Laser ablation of materials stands as a well-established technique extensively employed in laser-machining tasks, such as cutting, drilling, and microstructuring. These activities regularly take place in open-air environments, with a primary focus on manipulating the surface of the samples. In recent years, there has been a growing interest in employing ultrafast lasers for micro- and nanomanufacturing, which has evolved into a growing international field. An additional reason for laser machining gaining popularity is due to its ability work within liquid environments, aimed at improving control over surface quality and ablation efficiency.

The incorporation of a liquid medium offers several advantages, including reducing heat transfer to the samples and achieving clean treated surfaces. This has led to the widespread utilization of laser ablation in liquids (PLAL) in various applications, including laser cleaning and laser printing.

One distinctive feature of PLAL, differentiating it from traditional laser machining where the target surface is the primary output, is that in PLAL, the dispersed materials resulting from ablation in the liquid become the final product. Typically, during PLAL, a portion of the ablated material undergoes chemical transformations at the surface of the resulting nanoparticles, leading to the formation of defects like oxidized noble metal NP surfaces or NPs modified by oxygen vacancies [19]. These defects serve a critical role by providing surface charge, thereby ensuring electrostatic stabilization. Furthermore, these defects offer advantages in applications such as optics and catalysis. However, the primary mass of the colloids often retains the same chemical composition as the bulk targets, forming various materials, including alloys, binary compounds, and even ternary materials. Hence, PLAL is widely recognized as a hybrid method, involving elements of both top-down (involving macroscopic solid targets) and bottom-up (involving initially formed plasma, atoms, and clusters) approaches, with laser plasma and cavitation physics governing the initial processes [20].

**Figure 1:**
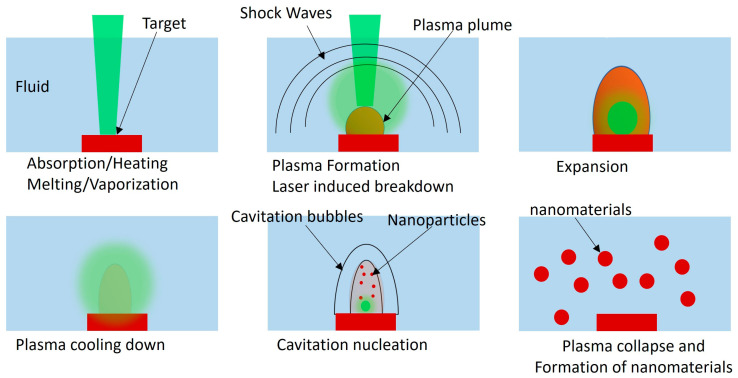
Schematic illustration of the laser ablation during the laser−target-liquid system for each laser pulse.

Laser ablation in a liquid environment is a complex process used for the selective removal of metal from a target through the application of laser energy within a liquid medium [20,21,22,23]. This mechanism unfolds in several stages as shown in Figure 1 [23], beginning with the absorption of the laser pulse by the metal, causing it to undergo rapid heating and subsequently vaporization. The vaporization process gives rise to the formation of plasma, which rapidly expands, generating shock waves that propagate through the liquid medium. These shock waves induce the formation of cavitation bubbles, which ultimately collapse, leading to the generation of high-pressure liquid streams directed towards the metal surface. The impact of these high-pressure liquid streams initiates the fracturing and ablation of the metal, resulting in the removal of material from the target.

The presence of plasma plays a pivotal role in enhancing the ablation process, as it not only absorbs a portion of the laser energy but also generates supplementary shock waves that further intensify cavitation and ablation. The efficiency of the laser ablation process within a liquid medium depends on multiple factors, including laser energy levels, laser wavelength, pulse duration, the nature of the liquid medium, and the inherent properties of the metal substrate. Optimization of these variables can lead to highly efficient and precise material removal, all while minimizing adverse effects on the surrounding material [1,22,24,25,26,27].

## 3. Au–Ag Bimetallic Nanoparticle Formation

Bimetallic nanoparticles consist of two different metals and can exist as alloys or core-shell structures. Until recently, the fabrication of bimetallic nanoparticles was limited due to limited methods available. In PLAL, diverse fabrication routes are explored. Typically, a monometallic colloid is first synthesized by an ablating target, like a Ag cube. This target is then replaced by another composed of a different metal, such as a Au cube, and ablated under the existing colloid to create Bimetallic nanoparticles, like Ag–Au bimetallic nanoparticles. This process often results in core–shell BNPs, where the core comprises the first ablated material and the shell consists of the second material. Smaller NPs (usually 1–5 nm) tend to be found on the shell, while larger particles (10–100 nm) form the core [28]. Another core–shell BNP formation method involves ablating a solid metal under a liquid medium containing metallic ions; the ions form the shells while NPs from the solid target create the cores. BNPs can also be produced by mixing two different metallic colloids and ablating the mixture, leading to either alloy or core–shell BNPs [28]. Alternatively, ablating two solid targets simultaneously results in alloy or core–shell BNPs. The composition ratios of the synthesized BNPs are influenced by laser parameters, laser wavelength, liquid medium type, and the metals being ablated. Since different metals have distinct optical and thermodynamic properties, their ablation efficiencies vary under the same laser parameters, further complicated by the different reactions of the liquid medium with the metals during BNP formation. A diverse range of bimetallic Au–Ag nanoparticles studies, obtained through the PLAL technique is highlighted in Table 1. Despite significant progress, there is still a need for more publications to optimize BNP formation through PLAL and comprehensively understand the factors leading to core–shell or alloy NP formation. Additionally, exploring the influence of laser parameters on the final chemical composition of BNPs remains an understudied area in the literature.

## 4. Parameters Influencing Au–Ag Bimetallic Nanoparticle Formation

### 4.1. Laser Wavelength

Numerous investigations have investigated the influence of laser pulse wavelength on the dimensions and yield of laser-ablated metal nanoparticles [36,37]. Regardless of other laser parameters, target, and solvent properties, it has been observed that employing infrared (IR) wavelengths results in the production of larger nanoparticles at a higher ablation rate compared to ultraviolet (UV) and visible wavelengths [19]. This phenomenon can be attributed to the fact that shorter wavelengths possess higher photon energies, thereby contributing to increased fragmentation. Conversely, longer wavelengths penetrate deeper into the target material, leading to a greater mass being ablated per pulse. However, when considering metal targets, the ablation rate is notably enhanced when visible or UV lasers are employed. This is due to the fact that, for most metals, absorption occurs in the UV range. Nevertheless, the synthesized metal nanoparticles absorb the incident laser pulse due to surface plasmon resonance (SPR), resulting in fragmentation and the formation of smaller nanoparticles [38,39]. Additionally, this absorption reduces the energy reaching the target surface, thereby diminishing the overall formation efficiency.

**Figure 2:**
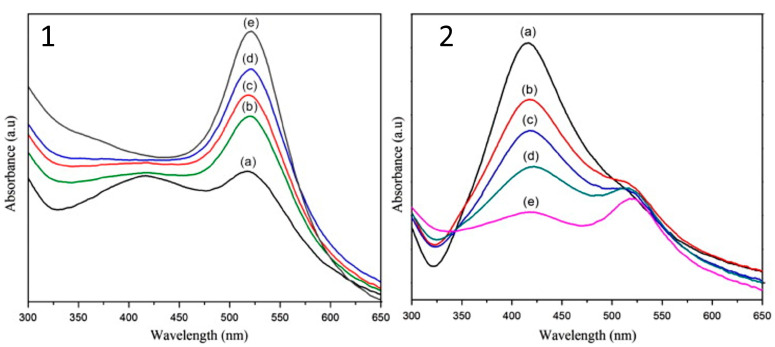
Absorption spectra of Au–Ag bimetallic nanoparticles prepared using a 355 nm laser (**1**) and a 1064 nm laser (**2**) for different ratios of Au and Ag nanoparticles, including (a) Au_20_:Ag_80_; (b) Au_40_:Ag_60_; (c) Au_50_:Ag_50_; (d) Au_60_:Ag_40_; and (e) Au_80_:Ag_20_. Reprinted with permission from reference [31]. Copyright 2014 Elsevier.

The synthesis of Au–Ag bimetallic nanoparticles using PLAL was investigated by Omar et al. [29] Gold and silver targets were individually subjected to laser irradiation using a Nd:YAG laser operating at a wavelength of 1064 nm and a pulse width of 8 ns. The zeta potential in the nanocomposite showed an increase in their negative surface charge compared to their individual component. Qayyum et al. [30] investigated the influence of 1064 nm and 532 nm lasers on the Au–Ag alloy formation. The alloying procedure was observed to be directly linked to the laser wavelength employed. Specifically, no alloy formation is found when employing a 1064 nm laser, whereas the utilization of a 532 nm laser is notably effective in facilitating alloy formation. However, the absorption spectra of Au–Ag bimetallic nanoparticles reported by Vinod et al. [31] reveal two distinct peaks representing the localized plasmon resonance of gold and silver nanoparticles as shown in Figure 2. Broadening of the gold nanoparticle plasmon resonance was observed with increased concentration of Au when Au/Ag mixing involved nanoparticles prepared at 355 nm, while no significant change occurred when nanoparticles ablated at 1064 nm were mixed. With increasing gold concentration, the plasmon resonance peak positions remained unchanged, confirming a mixture of separate Au and Ag nanoparticles without alloy formation. Conversely, for the synthesis at 1064 nm, the plasmon characteristics differed; as gold concentration increased, a broad plasmon band corresponding to Au emerged, indicative of a collective surface plasmon resonance involving both Au and Ag nanoparticles. This finding differs from the previously discussed result [30], highlighting the complex nature of pulsed laser ablation, in which the results are significantly influenced by material properties and the specific laser operational conditions.

### 4.2. Effect of Laser Energies

Laser fluence, expressed in J/cm^2^, signifies the energy distributed by the laser over a specific surface area. It relies on factors such as the beam diameter and the quantity and energy of photons within each pulse. A critical energy threshold marks the initiation of the ablation process. When the laser pulse energy is relatively low, the target absorbs the energy but does not eject material, and no plasma plumes form. Beyond this threshold, the yield of ablated nanoparticles exhibits an increase corresponding to the laser [22,40,41]. With higher laser fluence, the growth in absorption facilitates and amplifies the ablation rate. Additionally, at elevated laser fluence levels, the size of the ablated nanoparticles decreases. Excessive fluences causes the target surface to melt alongside the ablation process, resulting in reduced evaporation and self-laser light absorption. Consequently, this absorption-induced fragmentation leads to the generation of smaller particles [40]. Altowyan et al. [32] observed a minor size variation in Au–Ag metallic nanoparticles as they modified the laser ablation energy, while the structures of the nanoparticles remained unaffected. In another study, a minor LSPR wavelength shift was observed when employing the same 20 min exposure duration but varying irradiation fluences of 185, 139, and 99 mJ/cm^2^, resulting in LSPR wavelengths of 458 nm, 478 nm, and 496 nm, respectively [42]. Nguyen et al. [33] studied the synthesis of influence of Au–Ag bimetallic nanoparticles at different laser fluences (0.8, 1.2 and 1.6 W/cm^2^) during different irradiation times (10, 20 and 30 min). Prior to laser irradiation, there were distinct absorbance peaks observed at 516 nm and 421 nm, aligning with the individual SPR peaks of pure Au and Ag, respectively. However, upon exposure to laser irradiation at 532 nm, noticeable shifts in the absorption spectra occurred, showing alterations in the colloidal characteristics. Specifically, with a laser exposure duration of 10 min, a single absorption peak at 492 nm appeared when the average laser fluence increased to 1.6 W/cm^2^. Further, during both 20 min and 30 min laser irradiation periods, the characteristic SPR peak of the Au–Ag alloy nanoparticles became evident as the laser power density increased from 1.2 to 1.6 W/cm^2^. It is important to highlight that variations in laser fluence produce different outcomes depending on the material involved. For example, a study showed variations in responses to an increase in laser power of up to 200 µJ across a range of materials, including silver, titanium, cobalt, and steel [43].

### 4.3. Effect of Medium

**Figure 3:**
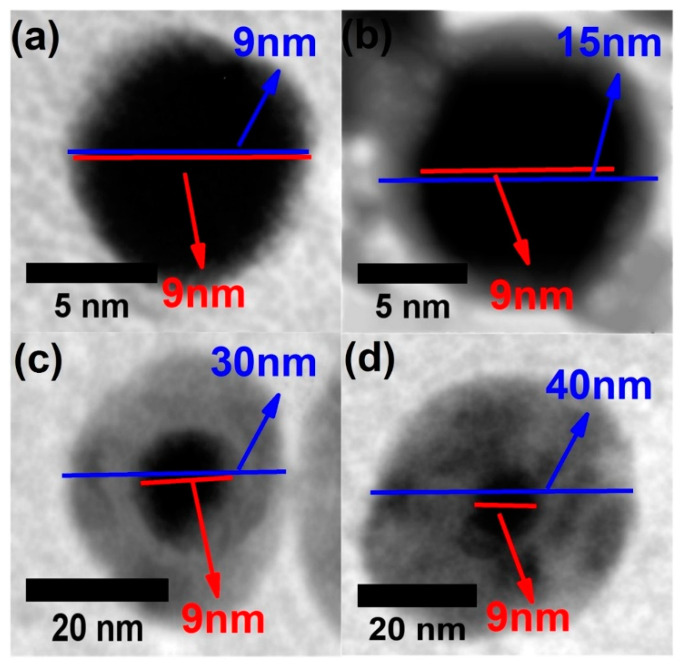
Transmission Electron Microscope (TEM) images of (**a**) silver nanoparticles (Ag NPs), and (**b**–**d**) Au–Ag core–shell bimetallic structures prepared at 1064 nm laser wavelength with varying shell thickness based on changes in chloroauric acid concentration from 0 mM chloroauric acid (**a**) to 1 mM chloroauric acid (**c**,**d**). The laser energy is 50 mJ for (**a**,**b**) and 100 mJ and 150 mJ for (**c**) and (**d**), respectively. Reprinted with permission from ref. [32]. Copyright 2021 Elsevier.

**Figure 4:**
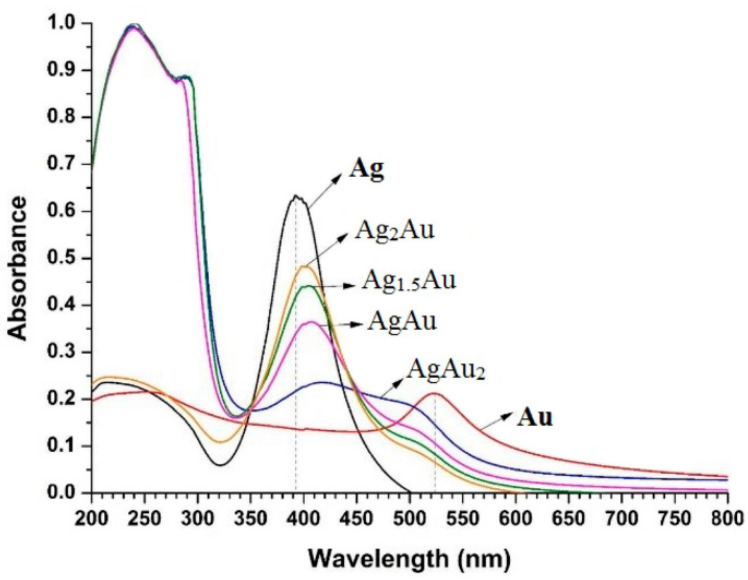
The UV-visible-near infrared (UV-Vis-NIR) absorbance spectrum of gold (Au) and silver (Ag) nanoparticles, alongside that of Au–Ag bimetallic nanoparticles (BNPs) following exposure to green laser irradiation. The presence of a peak at 523 nm corresponds to the dipolar Localized Surface Plasmon Resonance (LSPR) absorption of the Au nanoparticles (AuNPs). Additionally, another peak at 392 nm is attributed to the dipole LSPR absorption band of the Ag nanoparticles (AgNPs). The appearance of four dipole plasmon absorption bands (at 417 nm, 408 nm, 400 nm, and 397 nm) situated between the LSPR peaks of individual Au and AgNPs indicates the formation of bimetallic structures due to the plasmon decoupling of Au and AgNPs. Reprinted with permission from ref. [34]. Copyright 2023 Springer.

**Figure 5:**
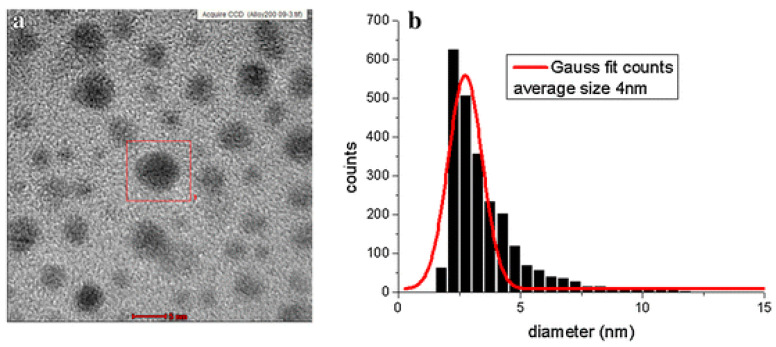
The TEM image (**a**) and the size distribution (**b**) for the Au–Ag bimetallic NPs. The laser wavelength and solvent used in this study are 532 nm and (Polyvinylpyrrolidone)n (PVP), respectively. Reprinted with permission from ref. [33]. Copyright 2015 Springer Nature.

The choice of liquid medium in which PLAL is performed stands out as a critical parameter that significantly influences various behaviors, including the distribution of nanoparticle sizes, their morphology, chemical properties, ablation efficiency, electrical conductivity, optical characteristics, and zeta potentials [28]. Altowyan et al. [32] examined how altering the solution used in the PLAL process of Ag plate, transitioning from ultra-pure water to ultra-pure water containing gold salt (1 mM chloroauric acid solution), see Figure 3. They attribute the shift from monometallic nanoparticles to bimetallic nanoparticles structured as core–shell configurations to changes in the surrounding medium accomplished through the utilization of a metallic precursor solution. It is worth noting that they studied the effect of laser energy simultaneously with the effect of the medium. In the study conducted by Adib Amini et al. [34], solutions with varying volumetric ratios were exposed to the second harmonic of the laser at a wavelength of 532 nm. This laser-induced synthesis process led to the formation of Au–Ag bimetallic nanoparticles. Figure 4 displays the outcomes of absorption spectra obtained from their nanocolloidal samples. These spectra show the plasmonic features exhibited by both monometallic nanoparticles (NPs) and bimetallic structures. An attempt was made by Chadha et al. to synthesize Ag–Au bimetallic nanoparticles within a medium containing 0.01 M SDS including 0.2 mM of Ag and Au nanoparticles [44]. The resultant particles exhibited a one absorption band at 520 nm, suggesting the solution was only composed of Au nanoparticles, with no sign of alloy-structure formation being detected due to the insufficient rates used in the generation. Homogeneous bimetallic Au–Ag nanoparticles, with an average diameter of 4 nm (see Figure 5), were successfully obtained through 532 nm laser irradiation within a Polyvinylpyrrolidone (C6H9NO)n (PVP) solution [33]. The choice of liquid medium in PLAL plays a pivotal role in influencing various aspects of the pulsed laser ablation process. Organic solvents like IPA, methanol, and ethanol, characterized by low boiling points, facilitate rapid liquid medium evaporation, making them advantageous for selected metal nanoparticles synthesis [45,46,47,48]. Notably, PLAL conducted in ethanol tends to yield larger NPs compared to DI water [36,49,50], while isopropyl alcohol has also been explored as a viable alternative [51]. Furthermore, previous PLAL investigations have explored the use of various organic solvents including toluene, tetrahydrofuran, and dimethylformamide to investigate their effects on the resulting NP size and morphology [52,53], Additionally, non-organic solvents like hydrogen peroxide have garnered attention [54]. It is important to emphasize that the specific choice of liquid medium exerts influence over colloidal density, UV-Vis absorbance, NP size distribution, and surface chemistry, given the differing interactions these liquids exhibit with the laser, the ablated material, and the synthesized NPs [28]. Hence, it is crucial to carefully choose the appropriate liquid medium, especially when synthesizing Ag–Au bimetallic nanoparticles in combination with other materials to create nanocomposites.

## 5. Biomedical Applications of Au–Ag Bimetallic Nanoparticles Synthesized by Pulsed Laser Ablation

### 5.1. Antimicrobial Applications

Silver nanoparticles (AgNPs) have garnered significant attention as potent antimicrobial agents in diverse applications like cosmetics, wound dressings, and dental materials [55,56]. While the exact antibacterial mechanism of AgNPs remains elusive, their antimicrobial efficacy is attributed to the release of silver ions (Ag+) and the generation of reactive oxygen species (ROS) [57,58]. Although AgNPs possess antimicrobial properties, indicating toxicity against microbial cells, they have also demonstrated toxicity towards various mammalian cells, revealing a lack of selectivity. This toxicity, arising from the high concentrations of released silver ions, severely affected the in vivo use of AgNPs. Previous research has indicated that encapsulating AgNPs with a gold shell can mitigate the release of Ag+ ions, enhancing their compatibility for in vivo applications. However, this gold coating diminishes the antibacterial efficacy of AgNPs, rendering the core–shell Ag–Au nanostructure inappropriate for the treatment of infectious diseases [59,60]. In contrast, integrating Ag with Au to create homogeneous Au–Ag nanoalloys (NAs) with an even elemental distribution substantially reduces the Ag+ release. The rate of release greatly depends on the composition of these two elements [61].

**Figure 6:**
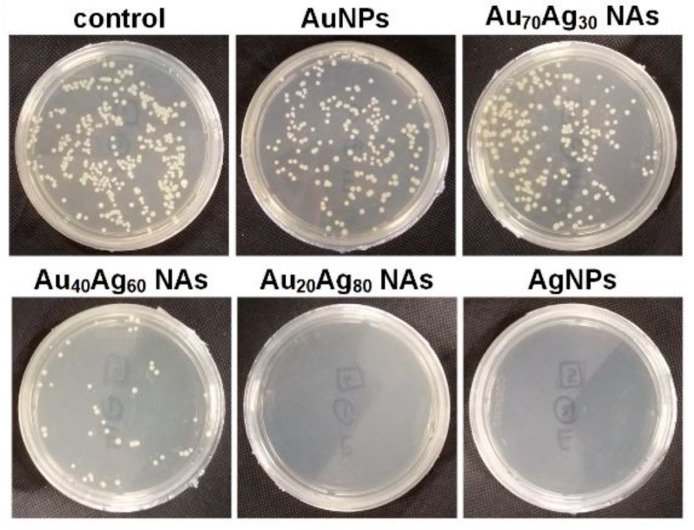
Plate counting analysis of E. coli after treatment with different Au–Ag bimetallic ratios. The study utilized a 1064 nm laser wavelength with a laser fluence of 45 J/cm^2^ in a water medium. Reprinted with permission from ref. [60]. Copyright 2021 Springer Nature.

Drawing from these findings, Lin et al. demonstrated that the alloying of Ag and Au at the atomic level remarkably mitigated the cytotoxicity associated with AgNPs [60]. A series of Au–Ag nanoalloys with varied elemental compositions were synthesized by pulsed laser ablation method. They manipulate the ratios of Au and Ag by adjusting the thickness ratio of ablated Au–Ag films. It was reported that nanoparticles containing 40% Au and 60% Ag exhibited substantial bactericidal effect with minimal cytotoxicity. The colony forming units (CFUs) of samples treated with pure AuNPs or Au_70_Ag_30_ bimetallic were comparable to the control samples, indicating the ineffectiveness of these nanoparticles in inhibiting bacterial colony formation. (see Figure 6). However, as the Ag atomic fraction increased to 60%, a notable decrease in CFUs was observed. When the Ag content exceeded 80%, almost no bacterial colonies were present on the plate. Further, Au–Ag bimetallic nanoparticles displayed lower cytotoxicity compared to their monometallic nanoparticle counterparts, attributed to reduced Ag+ release from the alloyed Au–Ag structures. Alkhayatt et al. employed a two-step approach using PLAL to produce bimetallic Au–Ag colloidal nanoparticles [62]. Initially, Au colloidal NPs were generated by laser-targeting high-purity gold plates placed at the bottom of a Pyrex container containing 2 mL of ultrapure deionized water. In the subsequent stage, the introduction of pure silver into the freshly produced gold colloidal NPs resulted in the formation of Au–Ag bimetallic NPs. The antibacterial efficacy of monometallic gold, silver, and bimetallic Au-Ag NPs (generated by laser ablation with varying pulse numbers of 100, 150, and 200 in deionized water) was evaluated against S. aureus and E. coli isolates. The inhibitory zone diameters indicated synergistic effects of the NPs on the tested bacteria, ranging from 4 mm to 15 mm at the lowest and highest values, respectively. Notably, this effect amplified with an increase in the number of laser pulses [63]. Contradicting the outcomes of previous studies, the process of alloying silver with gold through laser ablation in liquid yielded nanoparticles exhibiting diminished antibacterial and cytotoxic characteristics compared to pure silver nanoparticles [64]. Interestingly, this alloying strategy retained the combined application potential of both elements within a single colloid. These findings highlight the enhanced biocompatibility observed when silver was alloyed with gold, enabling bioconjugation through established thiol chemistry. However, this contradiction underscores the intricate nature of pulsed laser ablation, emphasizing its complexity in manipulating the properties of resulting nanoparticles.

### 5.2. Photothermal and Diagnostic Applications

Understanding heat transport mechanisms in nanoparticles is crucial for advancing nanotechnology applications. Precisely controlling heat transport at the nanoscale level is pivotal, opening new avenues for medical applications [65,66]. Current research on the thermal transport mechanisms of nanofluids primarily focuses on monometallic nanoclusters [67,68,69]. However, the ability to manipulate the composition of bimetallic nanoparticles offers promising prospects for enhancing thermal transport properties in comparison to their monometallic counterparts [70,71]. Bimetallic nanoparticles, explored extensively for enhancing thermal properties, hold immense potential in vital medical areas such as hyperthermia cancer treatments and targeted drug delivery systems. Further, these bimetallic nanoparticles find applications in photothermal therapy, where they absorb light and convert it into heat, destroying cancer cells selectively. Moreover, in hyperthermia, they raise the temperature of tumors to enhance the effectiveness of radiation therapy. Additionally, they play a crucial role in biosensing applications, enabling highly sensitive detection of biomolecules. The unique physicochemical characteristics of bimetallic nanoparticles appear from the synergy between two distinct metals and their altered electronic structures. Thermal diffusivity, a fundamental material property reflecting its responsiveness to thermal changes in the environment, plays a pivotal role [72,73]. Utilizing the highly sensitive thermal lens technique, a non-destructive method, plays a vital role in measuring thermal diffusivity, enabling the precise development of medical interventions.

Simon et al. synthesized bimetallic Au–Ag nanoparticles using a pulsed Nd:YAG laser operating at a fundamental wavelength of 1064 nm [66]. They subjected a silver metal plate to laser ablation for different durations in an existing colloidal solution of gold nanoparticles. Subsequent laser irradiation at 532 nm confirmed the formation of bimetallic nanoparticles, which was validated through meticulous analyses including Transmission Electron Microscopy (TEM) and absorption spectroscopy. Through thermal diffusivity studies using a dual beam mode matched thermal lens technique at different pump wavelengths, the researchers observed a systematic increase in thermal diffusivity values with prolonged ablation time of the silver plate in the gold nanoparticle solution. This increase was attributed to morphological variations in the bimetallic nanoparticles. Moreover, an increase in thermal diffusivity was noted with prolonged laser irradiation, indicating agglomeration of the bimetallic nanoparticles. The same group investigated the effect of concentration, morphology and composition on the thermal diffusivity using femtosecond laser ablation [74]. The thermal diffusivity values demonstrated a notable increase with higher sample concentrations and a rise in the Ag/Au weight percentage ratio. Particularly, the Au-Ag core-shell and nanoalloy structures exhibited significantly greater variations in thermal diffusivity compared to the other morphologies. At a constant laser input fluence, they observed a shift from saturable absorption (SA) to reverse saturable absorption (RSA) as the ablation time of the silver plate in the gold nanoparticle solution increased. A proportional increase in the nonlinear absorption coefficient with the escalation of input fluence was observed. The findings of these studies hold practical significance, especially in the application of plasmonic bimetallic nanoparticles as nanoheat sources in the medical field. Careful consideration should be given to the choice of liquid medium for synthesizing nanoparticles, especially with regard to their potential medicinal applications. It is always required to use solvents like aqueous solution and biocompatible polymer which is widely recognized for its biocompatibility and pharmaceutical safety.

Gold and silver nanoparticles demonstrate remarkable stability, making them promising candidates for delivery systems. Their high atomic numbers (Z = 79 for gold and Z = 47 for silver) and exceptional X-ray attenuation properties align with the requirements for effective X-ray contrast agents [75]. To create ideal contrast agents with minimal toxicity, it is essential to use pure nanoparticles. Khumaeni et al. synthesized Au–Ag bimettalic nanoparticles using pulsed laser ablation for in vitro and in vivo contrast enhancement [76]. The in vitro evaluation of combined Au–Ag nanoparticles exhibited superior contrast enhancement, outperforming other colloids with a remarkable 196% enhancement. The bimetallic of Au–Ag nanoparticles achieved the highest Hounsfield Units (HU) value at 302.

### 5.3. Conclusions and Outlook

The synthesis of Au–Ag bimetallic nanoparticles using pulsed laser ablations in liquid has made substantial contributions to diverse fields such as, energy production, optical imaging, catalysis, medical diagnostics, and beyond. However, several challenges remain. One major challenge is the limited productivity, which poses barriers in many laser synthesis labs and hinders the practical application of PLAL. The remarkable properties of these nanoparticles have primarily been applied in scenarios requiring minimal quantities due to their low production yield.

Analytical techniques like Raman spectroscopy, X-ray diffraction (XRD), Fourier transform infrared (FTIR), thermogravimetry, and application tests often require substantial quantities of nanoparticles, making it necessary to produce few grams quantities for statistical experiments. Further, optimization of various parameters such as scanning speed, repetition rate, pulse duration, focal settings, interaction with the surrounding liquid environment, and the geometry of the target material is a complex challenge, and it is worth noting that the optimal settings may differ based on the specific material, liquid medium, and pulse duration being employed. However, with the automation of PLAL, we anticipate a substantial improvement in their productivity. Automating the PLAL process presents a significant advancement. Automated systems outperform humans in terms of speed, and their ability to make real-time adjustments through feedback systems during fabrication is invaluable. Removing human intervention from tasks like beam focusing, liquid replenishment, and component cleaning is crucial. This not only minimizes errors that can occur due to human involvement but also saves essential production time, preventing interruptions that could otherwise lead to decreased output. While initial studies have explored remote monitoring and management of fabrication, higher levels of automation are imperative for the widespread commercial integration of this technology.

The technique of laser ablation became an attractive technique for fabricating to engineer bimetallic nanoparticles with enhanced properties. The rapid cooling rates and controlled environments inherent to PLAL facilitate the production of well-defined structures, ensuring uniformity and reproducibility in our samples. This level of precision is pivotal in medical applications where the nanoparticles’ properties directly impact their efficacy and safety. Further, PLAL enables the synthesis of nanoparticles directly in biocompatible liquid media, such as water, ensuring the absence of toxic residues or by-products. This intrinsic biocompatibility aligns well with medical standards, making PLAL-prepared nanoparticles inherently suitable for biological applications. Moreover, the uniform dispersion of these nanoparticles in liquid media is essential for consistent and controlled delivery within biological systems.

A comprehensive exploration of the links between the physico-chemical traits of nanoparticles and their biological impacts could pave the way for more sophisticated NP synthesis tailored to specific biomedical needs. To meet the demands of advanced in vivo diagnosis and therapy using functional nanoparticles, customized nanoparticles with exceptional properties can be crafted through modified pulsed laser ablation techniques. In particular, this represents the ultimate goal. These endeavors to enhance pulsed laser ablation conditions could facilitate the controlled large-scale production of diverse functional NPs, catering to the growing industrial interest and the prospect of commercialization across a wide spectrum of biomedical research and clinical applications in the future. Consequently, this advancement could significantly enhance the safe, sustainable utilization, and precision of systems grounded in PLAL, driving innovation in the PLAL technique.

## Figures and Tables

**Table 1:** Synthesis of various bimetallic Au–Ag nanoparticles via pulsed laser ablation in liquid.

Liquid Medium	Laser Wavelength	Laser Energy	Alloy/Core–Shell	Refs.
Di Water	1064 nm	55.2 J cm^−2^	core–shell	[29]
Di Water	1064 nm	0.4 J cm^−2^	no bimetallic formation	[30]
Di Water	532 nm	0.4 J cm^−2^	alloy	[30]
Di Water	355 nm	9.95–17.9 J cm^−2^	no bimetallic formation	[31]
Di Water	1064 nm	6.37–11.46 J cm^−2^	alloy	[31]
1 mM chloroauric acid solution	1064 nm	31–95 Jcm^−2^	core–shell	[32]
0.02 M PVP solution	532 nm	0.8, 1.2 and 1.6 W/cm^2^	Au–Ag alloy formation occurred at all selected laser fluences during 30 min of irradiation. At 20 min, alloys were formed at 1.2 and 1.6 W/cm^2^. At 10 min, alloys formed exclusively at 1.6 W/cm^2^.	[33]
HAuCl_4_,3H_2_O and AgClO_4_ at different molar ratio having 0.01 M SDS	532 nm	Not mentioned	no bimetallic formation	[34]
Di Water	532 nm	1.2 J cm^−2^	alloy	[35]

## Data Availability

Not applicable.

## References

[1] Alheshibri M., Kotb E., Haladu S.A., Al Baroot A., Drmosh Q., Ercan F., Çevik E., Elsayed K.A. (2023). Synthesis of highly stable Ag/Ta_2_O_5_ nanocomposite by pulsed laser ablation as an effectual antibacterial agent. Opt. Laser Technol..

[2] Zhang Y., He Z., Wang H., Qi L., Liu G., Zhang X. (2015). Applications of hollow nanomaterials in environmental remediation and monitoring: A review. Front. Environ. Sci. Eng..

[3] Ma J., Wei H., Liu Y., Ren X., Li Y., Wang F., Han X., Xu E., Cao X., Wang G. (2020). Application of Co3O4-based materials in electrocatalytic hydrogen evolution reaction: A review. Int. J. Hydrog. Energy.

[4] Zhang W., Yang S., Jiang M., Hu Y., Hu C., Zhang X., Jin Z. (2021). Nanocapillarity and Nanoconfinement Effects of Pipet-like Bismuth@Carbon Nanotubes for Highly Efficient Electrocatalytic CO_2_Reduction. Nano Lett..

[5] Augustyn V., White E.R., Ko J., Grüner G., Regan B.C., Dunn B. (2014). Lithium-ion storage properties of titanium oxide nanosheets. Mater. Horiz..

[6] Tian Q., Chen Y., Zhang W., Chen J., Yang L. (2020). Self-sacrificing template strategy to facilely prepare well-defined SnO2@C quasi-hollow nanocubes for lithium-ion battery anode. Appl. Surf. Sci..

[7] Yin Z., Huang R., Yu Y.-N., Ma W.-M., Cheng Y., Li Y.-B., He Y., Lv W.-Y., Cao L.-H. (2023). Multicomponent 3d-Metal Nanoparticles in Amorphous Carbon Sponge for Electrocatalysis Water Splitting. ACS Appl. Nano Mater..

[8] Zang Z. (2018). Efficiency enhancement of ZnO/Cu_2_O solar cells with well oriented and micrometer grain sized Cu2O films. Appl. Phys. Lett..

[9] Chattopadhyay K., Mandal M., Maiti D.K. (2021). Smart Metal-Organic Frameworks for Biotechnological Applications: A Mini-Review. ACS Appl. Bio. Mater..

[10] Ansari M.A., Albetran H.M., Alheshibri M.H., Timoumi A., Algarou N.A., Akhtar S., Slimani Y., Almessiere M.A., Alahmari F.S., Baykal A. (2020). Synthesis of Electrospun TiO_2_ Nanofibers and Characterization of Their Antibacterial and Antibiofilm Potential against Gram-Positive and Gram-Negative Bacteria. Antibiotics.

[11] Lee S.H., Ansah I.B., Mun C., Yang J.-Y., Shin S.-Y., Na J., Park J., Nam S.-Y., Lee S., Kim J. (2023). Galvanic engineering of interior hotspots in 3D Au/Ag bimetallic SERS nanocavities for ultrasensitive and rapid recognition of phthalate esters. Chem. Eng. J..

[12] Alwan A.M., Jawad M.F., Hashim D.A. (2019). Enhanced Morphological Properties of Macroporous Silicon with the Incorporation of Au-Ag Bimetallic Nanoparticles for Improved CO_2_ Gas Sensing. Plasmonics.

[13] Xia J., Chen G.Y., Li Y.Y., Chen L., Lu D. (2023). Rapid and sensitive detection of superoxide dismutase in serum of the cervical cancer by 4-aminothiophenol-functionalized bimetallic Au-Ag nanoboxs array. Front. Bioeng. Biotechnol..

[14] Ayodhya D., Sumalatha V., Gurrapu R., Babu M.S. (2023). Catalytic degradation of HIV drugs in water and antimicrobial activity of Chrysin-conjugated Ag-Au, Ag-Cu, and Au-Cu bimetallic nanoparticles. Results Chem..

[15] Rani P., Varma R.S., Singh K., Acevedo R., Singh J. (2023). Catalytic and antimicrobial potential of green synthesized Au and Au@Ag core-shell nanoparticles. Chemosphere.

[16] Sharma G., Kumar D., Kumar A., Al-Muhtaseb A.H., Pathania D., Naushad M., Mola G.T. (2017). Revolution from monometallic to trimetallic nanoparticle composites, various synthesis methods and their applications: A review. Mater. Sci. Eng. C.

[17] Khan I., Saeed K., Khan I. (2019). Nanoparticles: Properties, applications and toxicities. Arab. J. Chem..

[18] Abid N., Khan A.M., Shujait S., Chaudhary K., Ikram M., Imran M., Haider J., Khan M., Khan Q., Maqbool M. (2022). Synthesis of nanomaterials using various top-down and bottom-up approaches, influencing factors, advantages, and disadvantages: A review. Adv. Colloid. Interface Sci..

[19] Balachandran A., Sreenilayam S.P., Madanan K., Thomas S., Brabazon D. (2022). Nanoparticle production via laser ablation synthesis in solution method and printed electronic application—A brief review. Results Eng..

[20] Khairani I.Y., Mínguez-Vega G., Doñate-Buendía C., Gökce B. (2023). Green nanoparticle synthesis at scale: A perspective on overcoming the limits of pulsed laser ablation in liquids for high- throughput production. Phys. Chem. Chem. Phys..

[21] Zhang D., Gökce B., Barcikowski S. (2017). Laser Synthesis and Processing of Colloids: Fundamentals and Applications. Chem. Rev..

[22] Alheshibri M. (2022). Influence of Laser Energies on the Generation of Cobalt Oxide Nanoparticles via Laser Ablation in Liquid. Solid. State Phenom..

[23] Lam J., Amans D., Chaput F., Diouf M., Ledoux G., Mary N., Masenelli-Varlot K., Motto-Ros V., Dujardin C. (2013). γ-Al2O3 nanoparticles synthesised by pulsed laser ablation in liquids: A plasma analysis. Phys. Chem. Chem. Phys..

[24] Rawat R., Tiwari A., Arun N., Rao S.V.S.N., Pathak A.P., Tripathi A. (2020). Synthesis of CuO hollow nanoparticles using laser ablation: Effect of fluence and solvents. Appl. Phys. A.

[25] Mangababu A., Sarang Dev G., Chandu B., Bharati M.S.S., Venugopal Rao S., Nageswara Rao S.V.S. (2020). Structural investigations of picosecond laser ablated GaAs nanoparticles in different liquids. Nano-Struct. Nano Objects.

[26] Mintcheva N., Aljulaih A., Wunderlich W., Kulinich S., Iwamori S. (2018). Laser-Ablated ZnO Nanoparticles and Their Photocatalytic Activity toward Organic Pollutants. Materials.

[27] Alheshibri M., Akhtar S., Al Baroot A., Elsayed K., Al Qahtani H.S., Drmosh Q.A. (2021). Template-free single-step preparation of hollow CoO nanospheres using pulsed laser ablation in liquid enviromen. Arab. J. Chem..

[28] Nyabadza A., Vazquez M., Brabazon D. (2023). A Review of Bimetallic and Monometallic Nanoparticle Synthesis via Laser Ablation in Liquid. Crystals.

[29] Omar G., Abd Ellah R.G., Elzayat M.M.Y., Afifi G., Imam H. (2024). Superior removal of hazardous dye using Ag/Au core–shell nanoparticles prepared by laser ablation. Opt. Laser Technol..

[30] Qayyum H., Amin S., Ahmed W., Mohamed T., Ur Rehman Z., Hussain S. (2022). Laser-based two-step synthesis of Au-Ag alloy nanoparticles and their application for surface-enhanced Raman spectroscopy (SERS) based detection of rhodamine 6G and urea nitrate. J. Mol. Liq..

[31] Vinod M., Gopchandran K.G. (2014). Au, Ag and Au: Ag colloidal nanoparticles synthesized by pulsed laser ablation as SERS substrates. Prog. Nat. Sci. Mater. Int..

[32] Altowyan A.S., Mostafa A.M., Ahmed H.A. (2021). Effect of liquid media and laser energy on the preparation of Ag nanoparticles and their nanocomposites with Au nanoparticles via laser ablation for optoelectronic applications. Optik.

[33] Nguyen T.B., Nguyen T.D., Tran T.D., Thi T.H.N. (2015). Laser-Induced Synthesis of Au–Ag Alloy Nanoparticles in Polyvinylpyrrolidone (C_6_H_9_NO)_n_ Solution. J. Clust. Sci..

[34] AdibAmini S., Sari A.H., Dorranian D. (2023). Optical properties of synthesized Au/Ag Nanoparticles using 532 nm and 1064 nm pulsed laser ablation: Effect of solution concentration. SN Appl. Sci..

[35] Fazio E., Saija R., Santoro M., Abir S., Neri F., Tommasini M., Ossi P.M. (2020). On the Optical Properties of Ag-Au Colloidal Alloys Pulsed Laser Ablated in Liquid: Experiments and Theory. J. Phys. Chem. C.

[36] García Guillén G., Zuñiga Ibarra V.A., Mendivil Palma M.I., Krishnan B., Avellaneda Avellaneda D., Shaji S. (2017). Effects of Liquid Medium and Ablation Wavelength on the Properties of Cadmium Sulfide Nanoparticles Formed by Pulsed-Laser Ablation. ChemPhysChem.

[37] Hu S., Melton C., Mukherjee D. (2014). A facile route for the synthesis of nanostructured oxides and hydroxides of cobalt using laser ablation synthesis in solution (LASIS). Phys. Chem. Chem. Phys..

[38] Tsuji T., Watanabe N., Tsuji M. (2003). Laser induced morphology change of silver colloids: Formation of nano-size wires. Appl. Surf. Sci..

[39] Liang S.X., Zhang L.C., Reichenberger S., Barcikowski S. (2021). Design and perspective of amorphous metal nanoparticles from laser synthesis and processing. Phys. Chem. Chem. Phys..

[40] Kamali S., Solati E., Dorranian D. (2019). Effect of Laser Fluence on the Characteristics of Graphene Nanosheets Produced by Pulsed Laser Ablation in Water. J. Appl. Spectrosc..

[41] Mahdieh M.H., Fattahi B. (2015). Size properties of colloidal nanoparticles produced by nanosecond pulsed laser ablation and studying the effects of liquid medium and laser fluence. Appl. Surf. Sci..

[42] Kuladeep R., Jyothi L., Alee K.S., Deepak K.L.N., Rao D.N. (2012). Laser-assisted synthesis of Au-Ag alloy nanoparticles with tunable surface plasmon resonance frequency. Opt. Mater. Express.

[43] Hahn A., Barcikowski S., Chichkov B.N. (2007). Influences on nanoparticle production during pulsed laser ablation. J. Laser Micro Nanoeng..

[44] Chadha R., Sharma R., Maiti N., Ballal A., Kapoor S. (2015). Effect of SDS concentration on colloidal suspensions of Ag and Au nanoparticles. Spectrochim. Acta A Mol. Biomol. Spectrosc..

[45] Kudryashov S.I., Saraeva I.N., Lednev V.N., Pershin S.M., Rudenko A.A., Ionin A.A. (2018). Single-shot femtosecond laser ablation of gold surface in air and isopropyl alcohol. Appl. Phys. Lett..

[46] Zhao J., Zhang Y., Fang Y., Fan Z., Ma G., Liu Y., Zhao X. (2017). Synthesis of polyynes by intense femtosecond laser irradiation of SWCNTs suspended in methanol. Chem. Phys. Lett..

[47] Yogesh G.K., Shuaib E., Priya A.K., Rohini P., Anandhan S.V., Krishnan U.M., Kalyanavalli V., Shukla S., Sastikumar D. (2021). Synthesis of water-soluble fluorescent carbon nanoparticles (CNPs) from nanosecond pulsed laser ablation in ethanol. Opt. Laser Technol..

[48] Ismail R.A., Mousa A.M., Khashan K.S., Mohsin M.H., Hamid M.K. (2016). Synthesis of PbI2 nanoparticles by laser ablation in methanol. J. Mater. Sci. Mater. Electron..

[49] Moura C.G., Pereira R.S.F., Andritschky M., Lopes A.L.B., Grilo J.P.d.F., Nascimento R.M.D., Silva F.S. (2017). Effects of laser fluence and liquid media on preparation of small Ag nanoparticles by laser ablation in liquid. Opt. Laser Technol..

[50] Yang S., Cai W., Zhang H., Xu X., Zeng H. (2009). Size and structure control of Si nanoparticles by laser ablation in different liquid media and further centrifugation classification. J. Phys. Chem. C.

[51] Johny J., Sepulveda-Guzman S., Krishnan B., Avellaneda D., Shaji S. (2018). Facile and fast synthesis of SnS2 nanoparticles by pulsed laser ablation in liquid. Appl. Surf. Sci..

[52] De Bonis A., Curcio M., Santagata A., Galasso A., Teghil R. (2020). Transition Metal Carbide Core/Shell Nanoparticles by Ultra-Short Laser Ablation in Liquid. Nanomaterials.

[53] Xu X., Gao L., Duan G. (2018). The Fabrication of Au@C Core/Shell Nanoparticles by Laser Ablation in Solutions and Their Enhancements to a Gas Sensor. Micromachines.

[54] Scardaci V., Condorelli M., Barcellona M., Salemi L., Pulvirenti M., Fragalà M.E., Compagnini G. (2021). Fast one-step synthesis of anisotropic silver nanoparticles. Appl. Sci..

[55] Shakya S., He Y., Ren X., Guo T., Maharjan A., Luo T., Wang T., Dhakhwa R., Regmi B., Li H. (2019). Ultrafine Silver Nanoparticles Embedded in Cyclodextrin Metal-Organic Frameworks with GRGDS Functionalization to Promote Antibacterial and Wound Healing Application. Small.

[56] Chernousova S., Epple M. (2013). Silver as antibacterial agent: Ion, nanoparticle, and metal. Angew. Chem. Int. Ed..

[57] Xiu Z.M., Zhang Q.B., Puppala H.L., Colvin V.L., Alvarez P.J.J. (2012). Negligible particle-specific antibacterial activity of silver nanoparticles. Nano Lett..

[58] Zhang C., Hu Z., Deng B. (2016). Silver nanoparticles in aquatic environments: Physiochemical behavior and antimicrobial mechanisms. Water Res..

[59] Choi S., Han S.I., Jung D., Hwang H.J., Lim C., Bae S., Park O.K., Tschabrunn C.M., Lee M., Bae S.Y. (2018). Highly conductive, stretchable and biocompatible Ag–Au core–sheath nanowire composite for wearable and implantable bioelectronics. Nat. Nanotechnol..

[60] Lin Z., Luo Y., Liu P., Li Y., Yue J., Jiang L. (2021). Atomic-engineering Au-Ag nanoalloys for screening antimicrobial agents with low toxicity towards mammalian cells. Colloids Surf. B Biointerfaces.

[61] Siddiq A.M., Thangam R., Madhan B., Alam M.d.S. (2019). Counterion coupled (COCO) gemini surfactant capped Ag/Au alloy and core–shell nanoparticles for cancer therapy. RSC Adv..

[62] Alkhayatt A.H.O., Husain Moheel M., Malik Abood M. (2018). Antibacterial Activity of Mono and Bimetallic Au:Ag Colloidal Nanoparticles Prepared by Pulse Laser ablation PLA. J. Kufa-Phys..

[63] Amendola V., Meneghetti M. (2009). Laser ablation synthesis in solution and size manipulation of noble metal nanoparticles. Phys. Chem. Chem. Phys..

[64] Grade S., Eberhard J., Jakobi J., Winkel A., Stiesch M., Barcikowski S. (2014). Alloying colloidal silver nanoparticles with gold disproportionally controls antibacterial and toxic effects. Gold. Bull..

[65] Miao T., Ma W., Zhang X., Wei J., Sun J. (2013). Significantly enhanced thermoelectric properties of ultralong double-walled carbon nanotube bundle. Appl. Phys. Lett..

[66] Simon J., Anugop B., Nampoori V.P.N., Kailasnath M. (2021). Effect of pulsed laser irradiation on the thermal diffusivity of bimetallic Au/Ag nanoparticles. Opt. Laser Technol..

[67] Huang W., Qian W., El-Sayed M.A. (2005). Photothermal reshaping of prismatic Au nanoparticles in periodic monolayer arrays by femtosecond laser pulses. J. Appl. Phys..

[68] Zamiri R., Azmi B.Z., Husin M.S., Zamiri G., Ahangar H.A., Rizwan Z. (2012). Thermal diffusivity measurement of copper nanofluid using pulsed laser thermal lens technique. J. Eur. Opt. Soc..

[69] Sánchez-Ramírez J.F., Jiménez Pérez J.L., Cruz Orea A., Gutierrez Fuentes R., Bautista-Hernández A., Pal U. (2006). Thermal diffusivity of nanofluids containing Au/Pd bimetallic nanoparticles of different compositions. J. Nanosci. Nanotechnol..

[70] Toshima N., Yonezawa T. (1998). Bimetallic nanoparticles—Novel materials for chemical and physical applications. New J. Chem..

[71] Gutierrez Fuentes R., Pescador Rojas J.A., Jiménez-Pérez J.L., Sanchez Ramirez J.F., Cruz-Orea A., Mendoza-Alvarez J.G. (2008). Study of thermal diffusivity of nanofluids with bimetallic nanoparticles with Au(core)/Ag(shell) structure. Appl. Surf. Sci..

[72] Viswanathan A., Udayan S., Musfir P.N., Nampoori V.P.N., Thomas S. (2019). Enhancement of defect states assisted thermal diffusivity in solution-processed GeSeSb chalcogenide glass matrix on silver incorporation. J. Non Cryst. Solids.

[73] Ramya M., Nideep T.K., Nampoori V.P.N., Kailasnath M. (2019). Particle size and concentration effect on thermal diffusivity of water-based ZnO nanofluid using the dual-beam thermal lens technique. Appl. Phys. B.

[74] Simon J., Nampoori V.P.N., Kailasnath M. (2021). Concentration dependent thermo-optical properties and nonlinear optical switching behavior of bimetallic Au-Ag nanoparticles synthesized by femtosecond laser ablation. Opt. Laser Technol..

[75] Xi D., Dong S., Meng X., Lu Q., Meng L., Ye J. (2012). Gold nanoparticles as computerized tomography (CT) contrast agents. RSC Adv..

[76] Khumaeni A., Budi W.S., Avicenna S., Muniroh M., Kusumaningrum N., Damayanti O., Fitria S. (2023). Gold and silver nanoparticles as computed tomography (ct) contrast agents produced by a pulsed laser ablation technique: Study In-Vitro and In-Vivo. Rasayan J. Chem..

